# Nanopore-Sequencing Metabarcoding for Identification of Phytopathogenic and Endophytic Fungi in Olive (*Olea europaea*) Twigs

**DOI:** 10.3390/jof9111119

**Published:** 2023-11-18

**Authors:** Ioannis Theologidis, Timokratis Karamitros, Aikaterini-Eleni Vichou, Dimosthenis Kizis

**Affiliations:** 1 Laboratory of Toxicological Control of Pesticides, Scientific Directorate of Pesticides’ Control & Phytopharmacy, Benaki Phytopathological Institute, 8 St. Delta Street, 14561 Athens, Attica, Greece; i.theologidis@bpi.gr; 2 Bioinformatics and Applied Genomics Unit, Department of Microbiology, Hellenic Pasteur Institute, 127 Vasilissis Sofias Avenue, 11521 Athens, Attica, Greece; tkaram@pasteur.gr; 3 Laboratory of Mycology, Scientific Directorate of Phytopathology, Benaki Phytopathological Institute, 8 St. Delta Street, 14561 Athens, Attica, Greece; k.vichou@bpi.gr

**Keywords:** metabarcoding, high-throughput sequencing, nanopore sequencing, olive, plant pathogen, *Verticillium dahliae*, endophytes, microbiome analysis, taxonomic profiling

## Abstract

Metabarcoding approaches for the identification of plant disease pathogens and characterization of plant microbial populations constitute a rapidly evolving research field. Fungal plant diseases are of major phytopathological concern; thus, the development of metabarcoding approaches for the detection of phytopathogenic fungi is becoming increasingly imperative in the context of plant disease prognosis. We developed a multiplex metabarcoding method for the identification of fungal phytopathogens and endophytes in olive young shoots, using the MinION sequencing platform (Oxford Nanopore Technologies). Selected fungal-specific primers were used to amplify three different genomic DNA loci (ITS, beta-tubulin, and 28S LSU) originating from olive twigs. A multiplex metabarcoding approach was initially evaluated using healthy olive twigs, and further assessed with naturally infected olive twig samples. Bioinformatic analysis of basecalled reads was carried out using MinKNOW, BLAST+ and R programming, and results were also evaluated using the BugSeq cloud platform. Data analysis highlighted the approaches based on ITS and their combination with beta-tubulin as the most informative ones according to diversity estimations. Subsequent implementation of the method on symptomatic samples identified major olive pathogens and endophytes including genera such as *Cladosporium*, *Didymosphaeria*, *Paraconiothyrium*, *Penicillium, Phoma, Verticillium*, and others.

## 1. Introduction

High-throughput sequencing technologies have changed the way of studying plant pathogen diagnostics and the dynamics of microbial populations by providing ease in the use of massive datasets, depth of analysis, and wider span of related information to evaluate [1]. Metagenomics and metabarcoding approaches have been facilitated initially with the advent of Next-Generation Sequencing (NGS) platforms such as 454 pyrosequencing, Illumina, and Ion Torrent, followed by third-generation sequencing technologies developed by Pacific Biosciences (PacBio) and Oxford Nanopore Technologies (ONT) [2]. Technical differences between these technologies relate to the different chemistries applied and to ways of nucleotide sequence recording, but also include differences in throughput, read length, accuracy and data analysis pipelines used [3]. In parallel to the advances in sequencing technologies, there has been a continuous development and evolution of algorithmic tools regarding initial processing such as demultiplexing, denoising and chimera detection, and subsequent steps for annotation, and taxonomic and functional assignments [1,4]. Though there is no consensus for the data analysis approach regarding the different platforms, there are suggested pipelines according to the experiment’s objective set [5,6,7], with the taxonomic and functional assignments being highly dependent to the reference database(s) used [8,9] and to the selection of the appropriate DNA sequence targets specified contextually.

Early and accurate identification of plant pathogens using molecular approaches is of great importance for the in-time and efficient management of plant diseases [10]. Simplex and multiplex PCR or immunodetection assays, though highly specific, are limited to the number of previously specified targets to be detected. On the contrary, an untargeted or delimited massive metabarcoding sequencing approach could provide information for all pathogenic species—either principal or opportunistic—present in a plant tissue or organ, and, furthermore, could provide a broader image of the epiphytic and endophytic microflora also present. Such approaches give an advantage in plant pathogen diagnostics by determining the predominant pathogen(s), the relative abundance(s), and the equilibria with putative antagonistic microorganisms already inhabiting the plant tissues.

The Oxford Nanopore Technologies sequencing platform, on the contrary to existing NGS approaches, provides long-sequence raw reads that can be used without the need of a prior assembly step, giving the advantage of using anticipated proper full-length sequences for downstream mapping. Furthermore, thus far, ONT is the only platform currently supporting off-lab and in situ (field/point-of-care) sample preparation, sequencing, and on-line real-time data analysis, providing an advantage for in-field diagnostic applications and studies on population dynamics [11,12,13].

Nanopore sequencing has been used for phytodiagnostics of different pathogenic species and following different approaches. These include the detection of different plant viruses in potato [14], tomato [15], cassava [10], wheat [16], yam [17] and various other plants [15,18,19] either following standard MinION protocols or elaborated cost-effective adaptations [18,20]. Several relevant studies provide reports on the identification of *Xylella fastidiosa* subspecies and sequence types from naturally infected plant material [21], tomato phytopathogenic bacteria identification [22], and mango dieback disease-associated fungal species [23]. Furthermore, there are studies on the detection of selected plant pathogens spanning bacteria, fungi, viruses and a phytoplasma in artificially inoculated plants via genomic DNA or RNA sequencing accordingly [15], and detection of plant and insect pathogens via induced meta-transcriptome analyses [24].

Olive (*Olea europaea* L.) is a main crop cultivated within the Mediterranean region due to its local and historically high socioeconomic importance. It is putatively expanding cultivation on a global scale due to the growing awareness in olive oil’s consumption’s health benefits, and, furthermore, as a rich source (including drupes, olive oil, and olive drupe processing byproducts) of biologically active compounds valuable for food, cosmetic, and pharmaceutical industries [25,26,27]. Though moderately tolerant to abiotic stress such as drought and salinity [28], the olive tree is host for many pests and a variety of pathogenic microorganisms, mainly fungi, resulting in a multitude of diseases and consequent decrease in tree fitness, productivity, and oil quality [29]. Most widespread foliar diseases of fungal origin include olive leaf spot, anthracnose and cercosporiosis caused by *Venturia oleaginea*, *Colletotrichum* spp., and *Pseudocercospora cladosporioides*, respectively [30,31,32,33]. Common drupe fungal pathogens include *Colletotrichum gloeosporioides* and *Camarosporium dalmaticum* which cause olive fruit rot [34,35]. Two vascular tissue diseases are *Verticillium* wilt [36,37] and shoot necrosis [38,39], caused by *Verticillium dahliae* and *Phoma incompta*, respectively, with the former pathogen capable of being extremely devastating to olive trees. The symptoms of both diseases are similar to a certain extent and typical of soilborne diseases, including wilted leaves that remain attached, lesions on shoots, and twig necrosis. Other fungal pathogens belonging to the *Cladosporiaceae* and *Dothideaceae* families have also been reported to cause drupe rots or act as saprophytes of leaves, twigs, and branches [40,41].

Examples of available metabarcoding approaches for the characterization of fungal disease complexes and oomycetes in different crops include *Fusarium* species in maize and different cover crops [42,43], causative agents of grapevine trunk and foliar diseases [44,45,46,47], and fungal communities among which species of the *Cladosporium* and *Botrytis* genera in aerial organs of strawberry plants [48]. Furthermore, studies on the characterization of the fungal and oomycete communities in soil samples, and on reciprocal influences between pathogens and plant or soil microbiome communities are reported [49,50,51,52].

Currently, several metagenomics studies regarding the epiphytic and endophytic mycoflora of olive phyllosphere and carposphere are available. These span the analysis of fungal diversity under different phenological stages [53], composition of microbial epiphytical communities on olive drupe in relation to maturation process and genotype [25], phyllosphere endophytic mycobiome of different cultivars [26], and host–pathogen interactions in association with the phyllosphere mycobiome [27,54]. Similar approaches regarding spoilage microorganisms during fermentation [55], effect of salt stress on leaf endophytic bacterial communities of different cultivars [56], or the genotype effect on bacterial populations [57], provide data on the shapes of phyllosphere or carposphere bacterial communities. Furthermore, a metabarcoding approach based on genus-specific primers was developed for the characterization of *Colletotrichum* species complexes in different olive tissues [58].

The aim of this research was to develop and evaluate a nanopore sequencing metabarcoding method for olive pathogenic fungi identification, and mycoflora characterization. Our results show that the method, either as a multiplex or simplex approach, can efficiently identify diverse fungal communities that include main vascular tissue pathogens and olive endophytic fungi.

## 2. Materials and Methods

### 2.1. Sampling

Five apparently healthy olive twig samples, each one consisting of two to three twigs without any visual symptoms, were collected. From plants with visual symptoms ten samples were collected. Each of the ten samples consisted of two to three twigs with chlorotic (yellow) and dead brown leaves remaining attached on the apical part of twigs, and also containing apparently healthy green leaves. The samples were collected from separate olive trees in, respectively equal number of olive groves located in main olive cultivation regions of Attica, Crete, Central and Western Greece, Halkidiki, and Peloponnese from March to June 2019. Following collection, the samples were directly shipped to the laboratory overnight. From each sample, fine slices of tissues (approximately 3–5 mm in length) including both xylem and phloem were cut from 2 to 3 cm twig parts in regions delimited by green and chlorotic leaves, or approximately at similar longitudes regarding healthy twigs, using sterile scalpel, forceps, and plastic Petri dishes. Darkening of xylem tissue was not observed in any of the samples. Half of the slices were used for microbiological isolation and morphological characterization of fungal isolates, and the other half for DNA extraction.

### 2.2. Growth, and Morphological Characterization of Fungal Isolates

Sample tissue slices from the five healthy and ten symptomatic samples were transferred οn Potato Dextrose Agar (PDA) medium (BD Difco, Detroit, MI, USA) plates supplemented with 250 μg/mL Chloramphenicol (Sigma-Aldrich, Saint Louis, MO, USA), 5 μg/mL Penicillin G sodium salt (Sigma-Aldrich, Saint Louis, MO, USA), 30 μg/mL Streptomycin sulfate salt (Sigma-Aldrich, Saint Louis, MO, USA) antibiotics and incubated at 21 °C for up to ten days for isolation of fungi. Isolates were transferred to PDA and left to grow until sporulation at 21 °C. Subsequently, optical observation was performed under an Olympus CX22LED (Olympus, Waltham, MA, USA) microscope and characterization using morphological identification keys.

*Alternaria alternata* BPIC 2709, *Aspergillus niger* FMCC F112, *Cladosporium* sp., *Penicillium commune* FMCC F2, *Phoma incompta* BPIC 2668, and *Verticillium dahliae* BPIC 2696 isolates (available in the Benaki Phytopathological Institute Collection (BPIC) and the Agricultural University of Athens Food Microbiology Culture Collection (FMCC)) were used as reference in fungal-specific primer evaluation assays (Section 2.4) and were grown on PDA.

### 2.3. DNA Extraction

Plant tissue homogenization was performed in liquid nitrogen using sterile mortars and pestles. DNA extraction was performed with the innuPREP Plant DNA kit (Analytik Jena AG, Jena, Germany) according to the manufacturer’s instructions. DNA from fungal isolates used as reference was extracted with the NucleoSpin Microbial DNA Mini kit (Macherey-Nagel, Düren, Germany) according to the manufacturer’s instructions. The quantity and quality of purified DNA extracts were determined using an IMPLEN P330 Nanophotometer (Implen GmbH, München, Germany) and by electrophoresis on a 1.0% agarose gel.

### 2.4. Fungal-Specific Primers for Metabarcoding Assays

The Internal Transcribed Spacer and rRNA 28S large subunit primers ITS1Fngs, ITS4ngs, LR5 [59], and beta-tubulin Bt2a, Bt2b, and calmodulin CMD5, CMD6 [60] fungi-specific primers were selected for this study. To each of the ITS1Fngs, Bt2a and CMD5 primers, the 5′-TTTCTGTTGGTGCTGATATTGC-3′ tail-tag, and to each of the ITS4ngs, LR5, Bt2b and CMD6 primers, the 5′-ACTTGCCTGTCGCTCTATCTTC-3′ tail-tag, were added to the primers’ 5′ ends, respectively, according to instructions in the “Four-Primer PCR Barcoding protocol” provided by ONT. Thus, the resulting forward ont-ITS1Fngs (5′-TTTCTGTTGGTGCTGATATTGCGGTCATTTAGAGGAAGTAA-3′), ont-Bt2a (5′-TTTCTGTTGGTGCTGATATTGCGGTAACCAAATCGGTGCTGCTTTC-3′) and ont-CMD5 (5′-TTTCTGTTGGTGCTGATATTGCCCGAGTACAAGGAGGCCTTC-3′) and reverse ont-ITS4ngs (5′-ACTTGCCTGTCGCTCTATCTTCTCCTSCGCTTATTGATATGC-3′), ont-LR5 (5′-ACTTGCCTGTCGCTCTATCTTCTCCTGAGGGAAACTTCG-3′), ont-Bt2b (5′-ACTTGCCTGTCGCTCTATCTTCACCCTCAGTGTAGTGACCCTTGGC-3′) and ont-CMD6 (5′-ACTTGCCTGTCGCTCTATCTTCTCCCCGATAGAGGTCATAACGTGG-3′) primers were generated for use in metabarcoding assays.

The primer pairs were evaluated in PCR using DNA from different fungal species (Section 2.2). PCR was performed in a 20 μL reaction mixture (10× PCR buffer; 2 mM MgCl_2_; 300 μM dNTPs; 300 nM of each primer; 1,2 U Taq DNA polymerase (Thermo Fisher Scientific, Waltham, MA, USA)) using 30 ng of extracted fungal DNA. Cycling conditions were set in a MiniAmp Plus thermal cycler (Thermo Fisher Scientific, Waltham, MA, USA) at: 1 cycle initial denaturation at 94 °C for 3 min, 5 cycles (denaturation at 94 °C for 30 s, annealing at 55 °C for 30 s, elongation at 72 °C for 60 s), followed by 35 cycles (denaturation at 94 °C for 30 s, annealing at 68 °C for 30 s, elongation at 72 °C for 60 s), and a final extension step at 72 °C for 5 min.

### 2.5. Verticillium dahliae-Specific Amplification in Plant Tissues by PCR

*Verticillium dahliae* DNA-specific amplification in extracted plant tissue DNA samples was performed in a 20 μL reaction mixture (10× PCR buffer; 1.5 mM MgCl_2_; 300 μM dNTPs; 200 nM of each primer; 1,2 U Taq DNA polymerase) using 1 μg of sample’s extracted total DNA and the Vd19 (5′-CGGTGACATAATACTGAGAG-3′) and Vd22 (5′-GACGATGCGGATTGAACGAA-3′) forward and reverse primers of the species-specific RFLP marker DB19-DB22 genomic sequence [61]. Cycling conditions were set in a MiniAmp Plus thermal cycler (Thermo Fisher Scientific, Waltham, MA, USA) at 1 cycle initial denaturation at 94 °C for 4 min, 35 cycles (denaturation at 94 °C for 30 s, annealing at 54 °C for 30 s, elongation at 72 °C for 60 s), and a final extension step at 72 °C for 5 min.

### 2.6. DNA Library Preparation and Sequencing

DNA libraries were prepared using the PCR Barcoding Kit SQK-PBK004 (ONT plc., Oxford, UK) according to the Four-Primer PCR Barcoding protocol provided by ONT. A total of seven DNA libraries were prepared, each one corresponding to a single olive twig sample, and were sequenced in two individual runs. One library was used in the first sequencing run and the remaining six in the second run.

For each sample, 30 ng of template DNA, along with the selected forward and reverse fungi-specific primers (either ont-ITS1Fngs and ont-ITS4ngs, ont-Bt2a and ont-Bt2b or ont-ITS1Fngs and ont-LR5) tailed with specific tags, was used in simplex PCRs to selectively amplify fungal DNA. To each DNA template–primer pair combination, a specific Barcode primer (BP02 for the sample in the first sequencing run, and BP07-12 accordingly for the samples in the second sequencing run), the NEB LongAmp Hot Start Taq 2X Master Mix (New England Biolabs, Ipswich, MA, USA) and nuclease-free water (Thermo Fisher Scientific, Waltham, MA, USA) were then added to a final volume of 50 μL. Following PCR set-up, DNA amplification was performed in a MiniAmp Plus thermal cycler (Thermo Fisher Scientific, Waltham, MA, USA) using the following cycling conditions: 1 cycle initial denaturation at 94 °C for 120 s, 5 cycles (denaturation at 94 °C for 30 s, annealing at 52 °C for 30 s, elongation at 65 °C for 50 s (for ont-ITS1Fngs/ont-ITS4ngs, and ont-Bt2a/ont-Bt2b primers) or 90 s (for ont-ITS1Fngs/ont-LR5 primers)), 35 cycles (denaturation at 94 °C for 30 s, annealing at 62 °C for 30 s, elongation at 65 °C for 50 s (for ont-ITS1Fngs/ont-ITS4ngs, and ont-Bt2a/ont-Bt2b primers) or 90 s (for ont-ITS1Fngs/ont-LR5 primers)), final extension at 65 °C for 5 min.

Amplicons (45 μL) were mixed with 40 μL of Agencourt AMPure XP beads (Beckman Coulter, East Windsor, NJ, USA) in a 1.5 mL DNA LoBind tube (Eppendorf SA, Hamburg, Germany), and were purified with the aid of a rotator mixer, a magnetic rack and freshly prepared 70% ethanol. Amplicons were eluted from the air-dried magnetic beads in 10 μL of a solution containing 10 mM Tris-HCl pH 8.0 and 50 mM NaCl. 1 μL of each eluate was quantified using an IMPLEN P330 Nanophotometer (Implen GmbH, München, Germany) and checked by agarose gel electrophoresis. Equimolar amounts of amplicons with the same barcode were then pooled into a 1.5 mL DNA LoBind tube resulting in 100 fmol of pooled barcoded amplicons’ mix in a final volume of 10 μL. Adapter ligation was performed by adding to the pooled DNA the Rapid Adapter Mix (ONT plc, Oxford, UK) resulting in a final library volume of 11 μL. In the case of the 1st sequencing run the library comprises of amplicons from all three primer pairs tagged with BP02. In the case of the 2nd sequencing run, six libraries comprise of ITS1Fngs-ITS4ngs and Bt2a-Bt2b amplicons tagged with BP07 to BP12.

Sequencing runs were performed on R9.4 flow cells (ONT plc., Oxford, UK) connected to a MinION Mk1B device (ONT plc., Oxford, UK). Priming of each flow cell was performed with a buffer mixture of Flush Tether and Flush Buffer (ONT plc., Oxford, UK). The 11 µL of the final library were mixed with Sequencing Buffer and Library Loading Beads (ONT plc., Oxford, UK) prior to loading onto the flow cell in a dropwise fashion. Sequencing was operated by the MinKNOW vs. 3.3.2 and Guppy vs. 5.0.3 software (ONT plc., Oxford, UK) for the generation of fast5 files and for the basecalling of raw signals into DNA sequences in fastq format, respectively, setting sequence trimming and quality check parameters as default, with the minimum qscore = 7. The run was stopped after 48 h and generated data were binned in different folders as “passed” and “failed” sequences.

### 2.7. Data Analysis and Statistics

“Passed” fasta-formatted sequences were used in bioinformatic analyses using BLAST+ v. 2.12.0 [62] and the cloud platform BugSeq (https://bugseq.com/free, accessed 3 Ocotober 2023) [63]. The NCBI nt v5 (23 January 2022) database was used for sequence alignments and identifications.

BLAST+ was implemented in a Linux environment using *blastn* and GNU *parallel* commands with maximum target sequences set to 5. Outputs were sorted by BitScore and were parsed with custom scripts in R [64]. Only the best hit according to BitScore was stored for each query read. Distinct operational taxonomic unit (OTU) classes were defined by setting sequence diversity cut-offs based on relative evolutionary divergence median and interval values according to Li et al. [65]: reads yielding more than 95% identity to subjects were classified as species; from 90% to 95% were classified as genera; from 90% to 80% as families. Further data manipulation and microbiome analysis was conducted in R, mainly using *tidyverse* [66], *phyloseq* [67] and *microbiome* [68] packages.

Analysis with the BugSeq metagenomics classifier [63] involved fastp (v0.20.1) for read quality control using a minimum average read quality of Phred 7, a minimum read length of 100 bp, and the default low-complexity filter [69]. Reads were mapped with minimap2 (v2.17) [70] to NCBI database. Deduced alignments to the reference sequences were reassigned using Pathoscope (version 2.0.7) based on a Bayesian statistical framework and default parameters [71]. Finally, the lowest common ancestor of reassigned reads was calculated and inputted into Recentrifuge (v1.1.1), with the minimum required taxa set to 1 and generic input mode for summarization and visualization [72]. Quality control results were summarized with MultiQC with a custom configuration and Phred thresholds for bad quality data to 7 [73].

### 2.8. Data Availability

Fast5 raw sequence data files have been deposited at the NCBI Sequence Read Archive (SRA) under the accession number PRJNA1036004.

## 3. Results

### 3.1. Selection and Verification of Plant Material and Fungi-Specific Primers for Metabarcoding

Five apparently healthy and with no visual symptoms, and ten symptomatic (chlorotic-dead brown leaves, and dead apical vascular tissues) olive twig samples were initially tested for the absence or presence of common phytopathogenic fungi. Fungal isolates grown on culture media with and without antibiotics were characterized by microscopical observation and standard morphological identification keys. All healthy samples proved free of common olive vascular tissue phytopathogenic fungi such as *V. dahliae* and *P. incompta*. *V. dahliae* was isolated from four out of ten symptomatic samples, whereas from the rest different fungi including *Alternaria* sp., *Aspergillus* sp., *Cladosporium* sp., *Cycloconium* sp., *Phoma* sp., and *Penicilium* sp. were isolated (Table 1). Additionally, extracted DNA preps from all fifteen samples were also tested in an end-point PCR assay for verification of *V. dahliae* detection, using species-specific primers. The results confirmed the presence or absence of *V. dahliae* in all samples tested (Supplementary Figure S1). Based on these results, one healthy and five symptomatic samples were selected for downstream metabarcoding assays (Table 1). Of the five healthy samples (no visual symptoms and negative PCR result), the choice of the healthy one (sample code 3120) was arbitrary since all healthy samples complied with the criteria. From the ten symptomatic samples (with visual symptoms and infected with pathogenic fungi isolated), the selection of the five symptomatic samples was based principally in the presence/detection of main vascular tissue phytopathogens such as *V. dahliae* and *Phoma* sp. (sample codes 1669, 1778, 2179, 3100 and 3184).

Fungi-specific primers targeting the ITS region, large ribosomal RNA subunit, calmodulin and beta-tubulin loci were initially selected after bibliographic search. A primer pair was selected for each of the calmodulin and beta-tubulin loci. For the ITS and large ribosomal RNA subunit (ITS-28S LSU), three different primers were selected, a common forward-targeting the ITS region start, and two reverse-targeting the end of the ITS or rRNA LSU D3 regions, respectively. Two different twenty-two base-pair overhangs were added to the 5′ ends of the forward or reverse primers prior to their synthesis. The final primer lengths ranged from 37 to 46 base-pairs. The four primer-pair combinations were tested in end-point PCRs with template DNA from isolates of different fungal species. The ont-ITS1Fngs/ont-ITS4ngs, ont-ITS1Fngs/ont-LR5 and ont-Bt2a/ont-Bt2b primer pairs amplified all DNA targets (Supplementary Figure S2) and were selected for downstream metabarcoding assays. Calmodulin targeting primers amplified only the *Aspergillus niger* DNA, indicating a specific detection of this species only among all different fungi tested in the PCR, thus, it was not used further. The specific calmodulin CMD5, CMD6 primer-pair has been used for fungi detection [60]; however, it could be probably used better as a barcode for identification of species for the *Aspergillus* genus.

### 3.2. Metabarcoding Analysis of Healthy Olive Twigs

Of the five healthy samples, one (sample code 3120; Table 1) was selected for metabarcoding analysis in a multiplex target loci approach, using the ONT chemistry and platform. The extracted DNA from this sample was denominated Sample 0. The unique DNA library (L0) preparation was prepared as described in Section 2.5 and presented in Table 2, using fungal-specific primers targeting three regions in two different loci: (i) the entire ITS-28S LSU, the (ii) the ITS alone, and (iii) the beta-tubulin gene. A 48-h sequencing run (Sequencing Run 1; Table 2) was performed using one sixth (1/6) of the R9.4 flow cell and default parameters. Basecalling with the Guppy 5.0.3 software resulted in 112,792 reads, with a 9.44 average quality score, summing a total yield of 89.7 Mbases, as shown in the EPI2ME report provided by ONT (Supplementary Figure S3).

**Figure 1:**
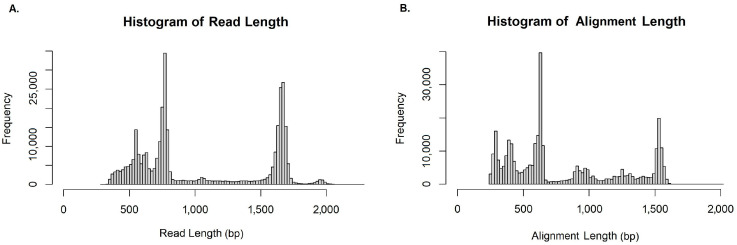
Distributions of lengths for raw reads and their BLAST+ matches for sample L0. Histograms depict the distributions of the lengths of (**A**) raw reads, and (**B**) their best matches according to BLAST+ analysis. Lengths smaller than 250 bp were excluded from panel (**B**). The leftward shift observed in the right panel (**B**) is the result of the exclusion of the adaptor sequences that total 160 bp in length.

The distribution of the lengths of raw reads (Figure 1A) exhibits three distinct peaks (at 540–560, 760–780 and 1680–1700 nucleotide bases, respectively) that correspond to the expected average PCR product sizes for the beta-tubulin, ITS and ITS-28S LSU loci [59,60,74]. Correspondingly, BLAST+ analysis yielded alignment of similar lengths (Figure 1B). However, since the distribution of the sizes does not distinguish between similar lengths originating either from truncated ITS, beta-tubulin or ITS-28S LSU products, a possible overlapping was expected. Therefore, two-fold filtering—by size but also by sequence descriptions—was necessary for the evaluation of locus combinations. Specifically, three bin sizes were assembled; one for beta-tubulin, ranging from 450 to 650 bp; a second for ITS, ranging from 650 to 850 bp; and a third one for ITS-28S LSU spanning from 1500 to 1750 bp. Size partitioning was driven by the rationale that bin margins should include the expected average amplicon sizes for each locus, and exclude alignments that correspond to truncated or chimeric sequences (region below 450 bp and region from 850 to 1500).

**Figure 2:**
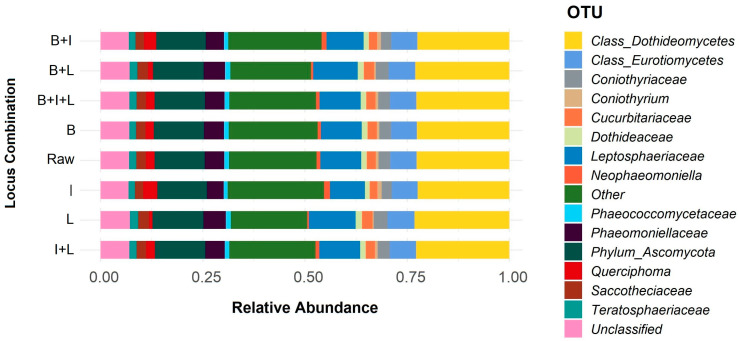
Relative abundance bar plots of annotated reads for loci combinations of Sample L0. Sample names refer to loci combinations after filtering by size and name. “Unclassified” OTUs correspond to alignments that lacked annotations at the taxonomic level of phylum or higher, while “Other” contain OTUs that were grouped together because they did not exceed the prevalence and detection thresholds (1% and 5%, respectively). B: beta-tubulin; I: ITS; L: ITS_28S LSU2; Raw: All loci without size and sequence description filtering. See Methods Section 2.7 for the determination of annotation cut-offs.

Two-fold filtering resulted in the relative abundance estimates shown in Figure 2 for sample L0. Annotated reads for all possible combinations regarding the three different loci are presented. Seven combinations comprise filtered annotated alignments and one represents all alignments obtained from the initial raw reads without any filtering. In all cases, the classification of annotations presented as operational taxonomical units (OTUs) up to the family level (see Methods and Figure 2 caption) include eight families (*Coniothyriaceae*, *Cucurbitariaceae*, *Dothideaceae*, *Leptosphaeriaceae*, *Phaeococcomycetaceae*, *Phaeomoniellaceae*, *Saccotheciaceae*, and *Teratosphaeriaceae*) and three genera (*Coniothyrium*, *Neophaeomoniella*, and *Querciphoma*) belonging to five fungal orders (*Pleosporales*, *Dothideales*, *Lichenostigmatales*, *Phaemonialles*, and *Mycosphaerellales*). Less than 10% of OTUs match to annotated entries that lacked any definition at the taxonomic level of phylum or higher (presented as unclassified), and nearly 14% belong to other families that did not exceed the prevalence and detection thresholds (1% and 5%, respectively). As observed, relative abundances are almost similar in all loci combinations.

**Figure 3:**
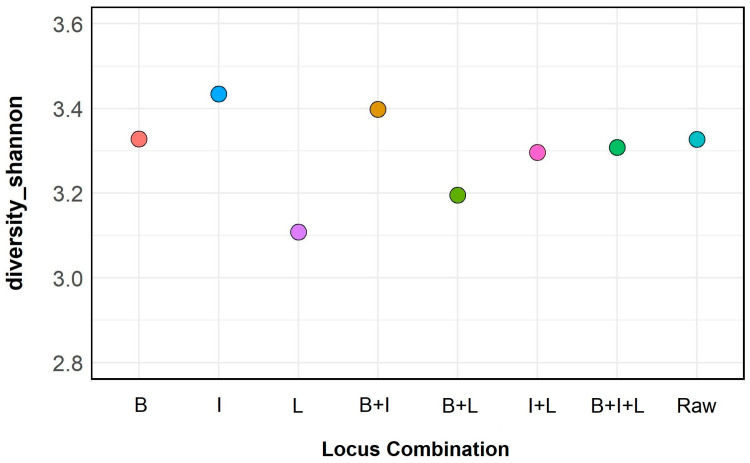
Alpha-diversity (Shannon Index, *H*) by locus combination in Sample L0. B: beta-tubulin; I: ITS; L: ITS_28S LSU; Raw: All loci without size and name filtering.

Το further gain insight on the content of Sample 0 and find the best combination of multiplexed loci regarding the diversity of the annotated reads, we proceeded to the calculation of the Shannon diversity index (*H—*Figure 3). As observed in Figure 3, ITS and the combination of beta-tubulin-ITS exhibit the higher *H* values, while ITS-28S LSU the lowest. The *H* value for beta-tubulin is similar to that of unfiltered annotations (Raw), with all other combinations following with lower values.

Combination of abundance estimates with diversity analysis indicate that a simplex ITS-targeting or a duplex (ITS and beta-tubulin combination) metabarcoding approach would constitute the best strategies for a detailed characterization of a biological sample for diagnostic schemes.

### 3.3. Metabarcoding Analysis of Symptomatic Olive Twigs

Following the initial evaluation of the metabarcoding method developed, we proceeded to its application in symptomatic olive twigs. In sequencing run 2 (Table 2), we used five new plant samples, along with the sample (healthy olive twigs) used in sequencing run 1, the DNA of which we priorly inoculated artificially with *V. dahliae* DNA. This spiked sample would serve as a control for sequencing run 2 and could also be compared to the original one used in sequence run 1. For the experiment, we generated libraries containing amplicons from both the ITS and the beta-tubulin loci.

A 48-h sequencing run (Table 2) was performed using one half (1/2) of the R9.4 flow cell and default parameters. Basecalling with the Guppy 5.0.3 software resulted in 4,081,641 reads, with a 9.98 average quality score, summing to a total yield of 2.6 Gbases as shown in the EPI2ME report provided by ONT (Supplementary Figure S4).

BLAST+ analysis of all reads was performed against the nt NCBI database (nt v5, 23 January 2022). Only one match per read was stored according to blast bitScore, constructing six distinct libraries. This process resulted in 3,745,679 annotated matches in total. For all six libraries, the distributions of the alignment lengths and the lengths of raw reads resulted in similar patterns (Supplementary Figure S5). Nevertheless, as in the previous case of Sample 0, filtering was performed at two levels: both by size and by description details.

**Figure 4:**
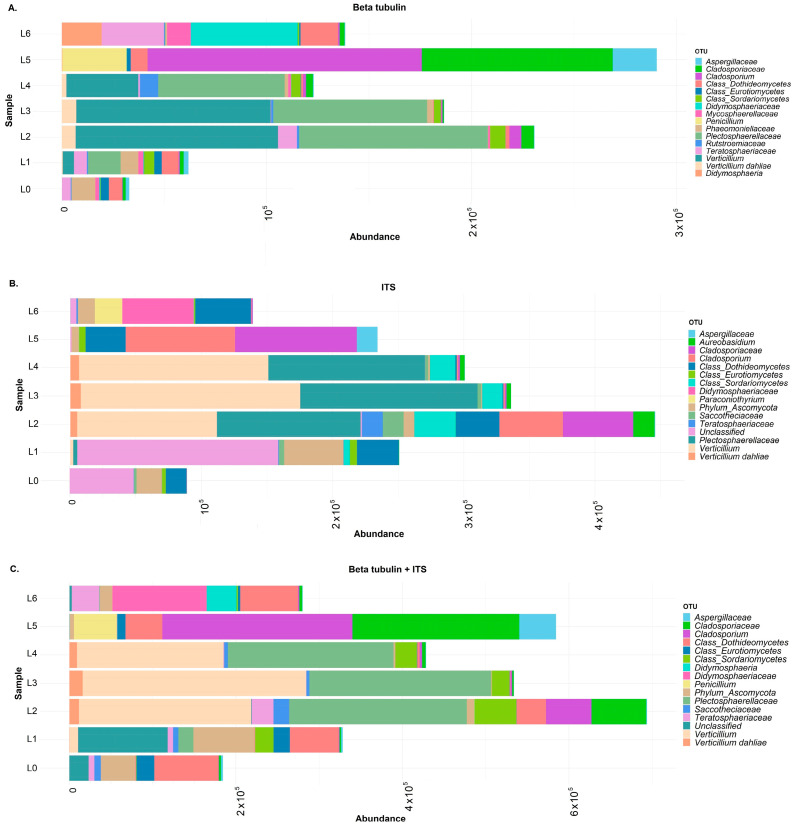
Absolute abundance bar plots of top-16 annotated OTUs for (**A**) beta-tubulin, (**B**) ITS, and (**C**) beta-tubulin + ITS. OTU: operational taxonomical unit; “Unclassified” OTUs correspond to alignments that lacked annotations lower than the “family” taxonomic level. See Methods Section 2.7 for the determination of annotation cut-offs. L0–L6: Library samples. Results regarding L0 from the 1st metabarcoding run are also presented for comparison with the L1 sample.

Absolute abundances of the annotated reads after filtering are presented for all three possible cases—(A) beta-tubulin, (B) ITS, and (C) ITS and beta-tubulin combined—regarding the two different loci used (Figure 4). In all cases, the classification of annotations is presented as operational taxonomical units (OTUs—see Methods and Figure 4 caption). For the single-plex approaches (ITS or beta-tubulin annotations only) differences are observed at the family level. Classification of beta-tubulin annotations (Figure 4A) results in eight families (*Aspergillaceae*, *Cladosporiaceae*, *Didymosphaeriaceae*, *Mycosphaerellaceae*, *Phaeomoniellaceae*, *Plectosphaerellaceae*, *Rutstroemiaceae*, and *Teratosphaeriaceae*) and four genera (*Cladosporium*, *Didymosphaeria*, *Penicilium*, and *Verticillium*), as well as for ITS (Figure 4B) in five families (*Aspergillaceae*, *Cladosporiaceae*, *Cucurbitariaceae*, *Didymosphaeriaceae*, and *Plectosphaerellaceae*), and five genera (*Cladosporium*, *Didymosphaeria*, *Paraconiothyrium*, *Penicilium*, and *Verticillium*), four of which are common with these in beta-tubulin classification. Regarding the duplex (ITS and beta-tubulin) approach, classification of annotations results in five family-level OTUs (four common in single-plex approaches and a new one (*Leptosphaeriaceae*)) and four genera, all of which are reported in both single-plex approaches (Figure 4C).

**Figure 5:**
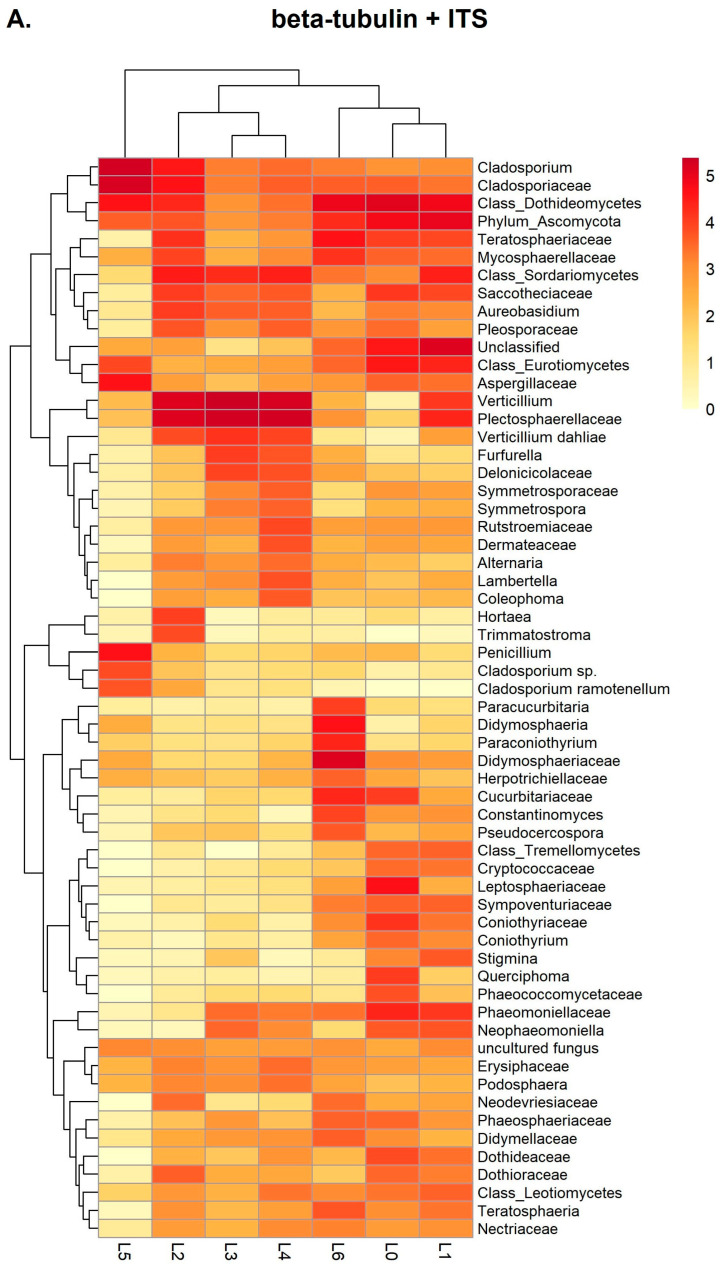

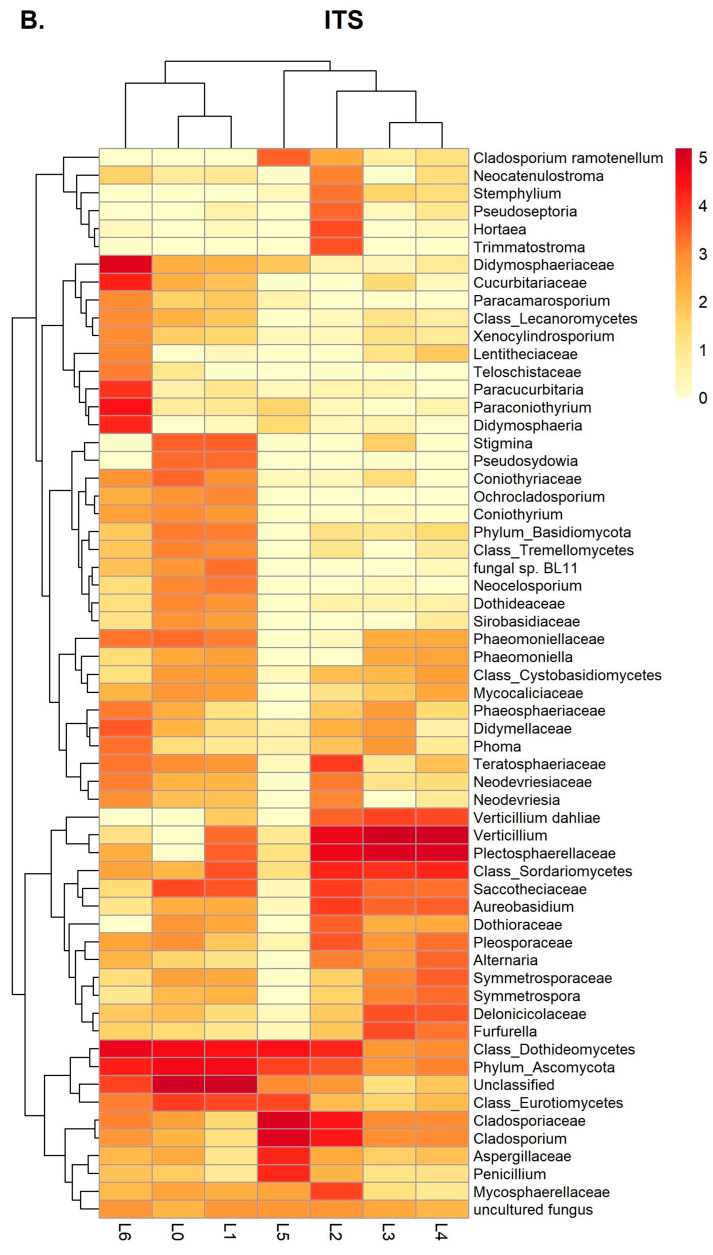

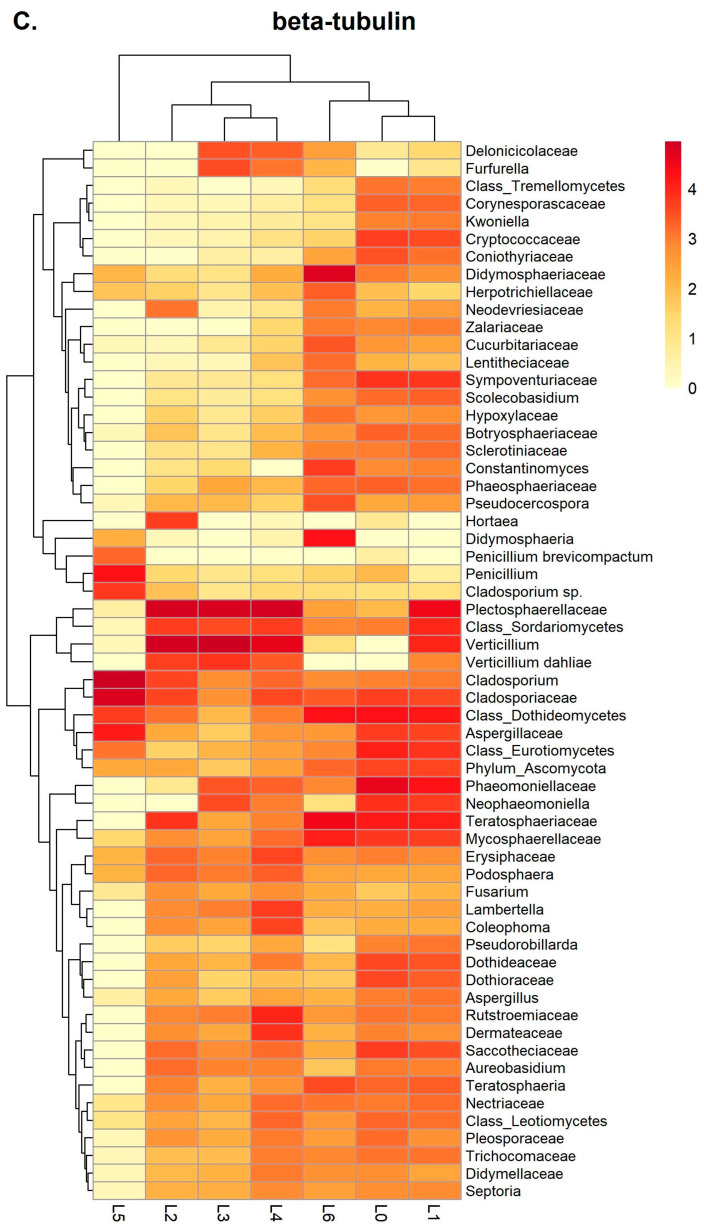
Heat-maps showing the top 60 OTUs down to species level for all samples (L0–L6) tested. (**A**) both ITS and beta-tubulin annotations are used. (**B**) ITS or (**C**) beta-tubulin single-locus annotations are used for OTU descriptions, respectively. L0–L6: Library samples. Libraries were normalized by median. Results regarding L0 from the 1st metabarcoding run are also presented for comparison with the L1 sample. Dendrograms represent compete linkage hierarchical clustering of samples and OTUs according to the normalized abundances.

A heat map presentation of the top 60 OTUs down to species level (that originate from combined ITS and beta-tubulin annotated OTUs) for all the samples tested is shown in Figure 5A. Of the twenty-one genera reported, eleven are predominant in samples L2–L6 with normalized log10 scale values higher than 4. Principal genera reported include *Cladosporium*, *Didymosphaeria*, *Hortea*, *Neophaeomoniella*, *Paraconiothyrium*, *Paracucurbitaria*, *Penicillium*, *Pseudocercospora*, *Querciphoma*, *Teratosphaeria*, and *Verticillium*. In each individual sample, the results for predominant genera identified conform with the results regarding morphologically characterized isolates reported in Table 1**.** *Verticillium* is highly represented in samples L2–L4 with classification going down to the species (*V. dahliae*) level. In samples L0 and L1, the difference regarding *Verticillium* spp is observed, as expected due to the artificial inoculation of sample L0 with *V. dahliae*. *Cladosporium* genus is present in relatively high numbers in all six samples with the highest representation in sample L5. Furthermore, *C. ramotenellum* is the second species-level OTU reported, having a high representation principally in sample L5. Sample L5 is also rich in annotations regarding *Penicillium* genus and species belonging to *Aspergillaceae* family. In sample L6 the *Didymosphaeria*, *Paraconiothyrium*, *Paracucurbitaria*, *Pseudocercospora* and *Teratosphaeria* genera along with annotations belonging to *Didymosphaeriaceae* family are highly represented. Equivalent heat map representations of OTUs when the ITS or beta-tubulin annotations are considered separately for analysis are shown in Figure 5B,C.

Common OTUs and differences observed at the genus and species levels regarding the three approaches (either separate loci or their combination), followed by data analysis, are presented collectively in Table 3.

For ITS-relevant annotations, there are 25 and 2 OTUs reported at genus and species levels, respectively. For beta-tubulin, there are 19 OTUs representing genera and 2 OTUs referring to species. For the 39 genera and 3 species reported collectively in Table 3, only 7 genera (*Aureobasidium*, *Cladosporium*, *Didymosphaeria*, *Furfurella*, *Hortaea*, *Penicillium* and *Verticillium*) are reported in both ITS and beta-tubulin OTUs, and are also present in OTUs when both loci annotations are counted for classification. Of the rest of the OTUs presented in Table 3, 18 and 13 genera are reported only in ITS or beta-tubulin OTU groups, respectively. When both loci annotations are counted the number of OTUs raises to 22, including the 7 common-in-all ones, 7 being also reported for ITS (*Alternaria*, *Coniothyrium*, *Paraconiothyrium*, *Paracucurbitaria*, *Stigmina*, *Symmetrospora*, and *Trimmatostroma)*, 7 being also reported for beta-tubulin (*Coleophoma*, *Constantinomyces*, *Lambertella*, *Neophaemoniella*, *Podosphaera*, *Pseudocercospora*, and *Teratosphaeria*), and one (*Querciphoma)* reported only in this group. Interestingly, there are OTUs at genus level reported either in the ITS group (*Neocatenulostroma*, *Neocelosporium*, *Neodevriesia*, *Ochrocladosporium*, *Paracamarosporium*, *Phaemoniella*, *Phoma*, *Pseudoseptoria*, *Pseudosydowia*, *Stemphylium*, and *Xenocylindrosporium*), or the beta-tubulin group (*Aspergillus*, *Fusarium*, *Kwoniella*, *Pseudorobillarda*, *Scolecobasidium*, and *Septoria*) which are not reported at all when both loci annotations are counted for OTU classification.

### 3.4. Data Analysis Using the BugSeq Platform

**Figure 6:**
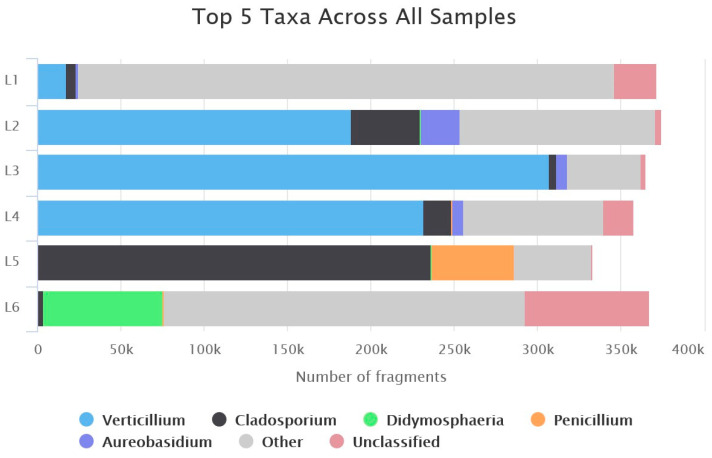
Bar plots of top five OTUs deduced from ITS + beta-tubulin raw reads’ analysis with the BugSeq platform. L1–L6: Sample libraries. The fragment numbers represent absolute reads for each sample.

Raw reads regarding samples L1–L6 were subjected to quality control, filtering, and analysis using the BugSeq pipeline, resulting in 2.285 M mapped reads. Data analysis for OTU determination was only possible for raw reads from the combination of both loci, since further filtering manipulations (i.e., according to sequence size and/or description) are currently not as available as online options. A classification of the top five genera for each library is presented in Figure 6. Predominant genera include *Verticillium* in samples L1–L4, *Cladosporium* particularly in L5 and, present in all samples, *Penicillium*, *Didymosphaeria* and *Aureobasidium*. The classification comes in agreement with the BLAST+ results presented in Figure 4C and Figure 5A.

## 4. Discussion

### 4.1. Approach Definition, Selection of Plant Material and Verification of Fungal-Specific Primers

High-throughput sequencing technologies have changed the way of studying plant and soil microbial population dynamics by providing ease in the use of massive datasets, depth of analysis, and wider span of related information to evaluate. They have also upgraded plant pathogen diagnostics and enabled studies on the equilibrium and interactions of pathogenic microorganisms with the remaining microbial flora present in a plant tissue [1,4]. In our study, we aimed at the development of a metabarcoding approach that could serve as method for a broad and untargeted identification of phytopathogenic and endophytic fungi present in olive young vegetative tissues.

The MinION platform (ONT plc., Oxford, UK) was chosen for the development of such a method since it is a third-generation sequencing technology that allows long-sequence reads, thus omitting the need for concatemeric short-read assembly prior to mapping, avoiding biases due to sequence gaps or presence of conserved non-species-specific sequences, and allowing direct mapping of full-length sequences [75]. Furthermore, nanopore sequencing is the only high-throughput sequencing technology that allows on-site application using portable devices, and real-time monitoring and evaluation of results [76,77]. The SQK-PBK004 PCR Barcoding Kit (ONT plc., Oxford, UK) was selected for our approach since it provides the ability to combine selective sequence amplification of a defined target group (in our case, fungi), multiplexing of several barcode sequences to amplify from the specific target group, and multiplexing of different samples/libraries using additional predefined barcodes.

Specific barcodes for fungi and oomycetes including the internal transcribed spacer (ITS), beta-tubulin, translation elongation factor-I (TEF-I) and RNA polymerase beta subunit (RPB-II) have been developed for the identification of such phytopathogens [78,79]. We selected four fungal-specific primer pairs previously reported in the relevant bibliography (see Section 2.4), targeting the Internal Transcribed Spacer (ITS), rRNA 28S large subunit, beta-tubulin, and calmodulin loci. The primers were modified by adding specific tail-tags, making them suitable for use in nanopore sequencing and were tested for amplification efficiency using genomic DNA from different phytopathogenic fungi isolates. All primer pairs but one (that targeting calmodulin) enabled amplicon production of the expected molecular weights from all fungal isolates, with calmodulin primers being specific only for *Aspergillus niger* (Supplementary Figure S2), indicating a putative future use of the specific locus region as a barcode for the genus *Aspergillus*. Thus, for subsequent experiments, we proceeded with three primer pairs targeting the ITS/rRNA 28S and beta-tubulin regions.

The inhouse-maintained NCBI’s nt database was selected for the workflow of the annotation of reads, as this study opts for the general inclusion of the maximum amount of sequence information. It is assumed that issues concerning maintenance and curation of this specific database and their impact on the outcome would be mitigated by the fact that only the best hits were used per read. Best hits were determined by the BLAST BitScore, which measures sequence similarity independent of database size, thus providing a constant statistical indicator for searching different databases of different sizes or for searching the same database at different times as the database enlarges.

Collection of olive twig samples was based on visual symptoms typical of vascular diseases caused by principal phytopathogens such as *V. dahliae* and *P. incompta* [36,38], and on apparently symptomless plants. The phytosanitary state of the samples, either diseased or healthy, was verified by classical microbiological and microscopy methods resulting in the isolation of various fungi including main phytopathogens such as *Cycloconium oleaginum*, *Phoma* sp., and *V. dahliae*, and opportunistic ones such as *Alternaria* sp., *Aspergillus* sp., *Cladosporium* sp., and *Penicilium* sp., as presented in Table 1. Identification of the isolates was based on morphological characteristics of spores, fruiting bodies, and fungal reproductive structures along with the use of taxonomical keys, and it was limited principally to the genus level.

Besides the aim of our work to develop a metabarcoding approach for a broad-spectrum identification of fungal phytopathogens and mycoflora, we chose to focus on *V. dahliae* as the principal phytopathogen to use as a reference species in our method. *V. dahliae* is a pathogen with a broad host range (nearly 400 species are affected), a remarkable survival capability in the soil, and is difficult to control since no effective chemical treatment exists. *Verticillium* wilt is a major soilborne disease for olive culture, affecting practically all cultivars. The symptoms of the disease are alike to those caused by *P. incompta*, another soilborne phytopathogenic species affecting vascular tissues causing branch dieback [37,39,80]. For this purpose, we verified *V. dahliae* presence or absence from the samples collected using species-specific primers in PCR (Supplementary Figure S1). Of the fifteen plant samples tested, we selected a healthy one (3120), and five (1669, 1778, 2179, 3100 and 3184) naturally infected with different phytopathogens (Table 1) for metabarcoding experiments.

### 4.2. Initial Set Up of the ONT Fungi-Specific Multiplex Metabarcoding Method

For the initial set up of the metabarcoding method, we used a healthy olive sample (3120) and tested different combinations of the three primer pairs targeting different loci or regions (ITS/rRNA 28S) in the same locus. A healthy tissue could provide a more generalized and representative background regarding the mycoflora equilibrium in a specific tissue when a diversity index is required. Instead, an infected tissue where the quantitative representation of the phytopathogen(s) are elevated could result in a shift towards certain species [42]. On the other hand, a multiplexed approach regarding sequence information originating from different loci/locus regions could possibly be more informative and precise regarding species annotation and phylogenetic relationships. Similar approaches such as Multi Locus Sequencing Analysis (MLSA) or MLSA combined with metagenomics are frequently used for the characterization of species and phylogenetic studies [81].

We generated a unique Library (L0) consisting of equimolar amplicons of the three different primer pairs used with the same sample’s (3120) genomic DNA (Table 2), which we sequenced using an R9.4 flow cell connected to a Mk1B MinION device (ONT plc., Oxford, UK). The R9.4 flow cell technology characteristics allow a reduced error rate compared to previous ONT flow cell types, with an observed read accuracy of nearly 98% [82]. Currently, the release of the ONT R10.4 flow cell allows a modal read accuracy of over 99.1% [82]; however, by the time the present research was conducted, R9.4 was the only flow cell type available. Basecalling resulted in approximately 113K reads, with an average quality score of 9.44, which is higher than the default cut-off limit of 7 (Supplementary Figure S3).

The three distinct peaks (at approximately 540–560, 760–780 and 1680–1700 bp) observed in the graphical presentation of the raw reads’ distribution (Figure 1A) confirmed the anticipated theoretical PCR product sizes for the three different loci, as also observed in agarose gel electrophoresis runs of the individual PCR products in Supplementary Figure S2. A histogram of the alignment lengths distribution after BLAST+ analysis (Figure 1B) depicted a similar pattern to this of raw reads. However, additional peaks for alignments of reads of smaller (<500 bp) and intermediate (approximately 900 to 1200 bp) sizes indicated alignments that might originate putatively from truncated or not full-length PCR products. Since the advantage of ONT sequencing for optimal annotation lies on the availability of full-length basecalled reads, we proceeded to further filtering of the aligned reads according to size (three bins of 450 to 650, 650 to 850 and 1500 to 1750 bp) based on the average loci sizes and sequence descriptions (beta-tubulin, ITS, and rRNA 28S LSU).

Following data filtering of the annotated reads, the relative abundance estimates were calculated for individual loci (three cases), their combinations (four cases), including also the unfiltered (raw for all loci) alignments. Differences between bar plots regarding the major OTUs up to the family level (eight families and three genera) are shown in Figure 2. Since there is a high similarity observed between the eight cases regarding abundances of the majority of the OTUs presented, and only subtle visual differences are seen, we proceeded to the calculation of the Shannon diversity index to depict the case(s) in which the highest possible diversity is achieved. As seen in Figure 3, the highest value is observed for annotations based on ITS, followed by its combination with beta-tubulin. Although the rRNA 28S LSU could theoretically provide more chances for differentiation between species due to its larger sequence length compared to ITS, it is important to take in consideration that the diversity outcome is also dependent on the number of annotations already available in a database that is implemented for the identifications/annotations. ITS sequence entries in NCBI are by far the most abundant compared to these of beta-tubulin and 28S LSU. It should also be noted that loci that show high variability in certain regions, such as the Intergenic Spacer (IGS) [83], may be good candidates for nanopore sequencing metabarcoding assays [84]. Based on the Shannon diversity index outcome, we decided to use ITS and beta-tubulin as targets for the subsequent metabarcoding assays.

### 4.3. Validation of the Metabarcoding Method on Infected Olive Twigs

For the metabarcoding analysis of symptomatic olive twigs, we used five samples (L2–L6) as reported in Table 2. We also used sample L0 that was tested in the first run, spiked with 1 ng DNA from *V. dahliae* to use as control (L1) in the current run. Like the outputs for the first run, a basecalling average quality score of 9.98 and similar patterns regarding the distributions of raw reads and alignment lengths (Supplementary Figures S4 and S5) were taken in consideration regarding the assay’s verity, prior to application of filtering parameters for BLAST+ analysis.

Classifications of absolute abundances for the single plex and duplex approaches (Figure 4A–C) show that predominant genera (*Cladosporium*, *Didymosphaeria*, *Penicilium*, and *Verticillium*) are common irrespective of the approach; however, that is with an additional genus (*Paraconiothyrium*) identified only in the ITS case. As mentioned previously, this may probably be anticipated since there is a larger number of ITS than beta-tubulin entries regarding this genus in the NCBI database. This results in a higher probability for query sequences to be classified as OTU at the genus level in the former case and family and broader level in the latter case. It is generally evident that uninfected sample (L0), or spiked sample (L1) libraries contain more OTUs that represent low-frequency classes (“Other”). Moreover, pathogenic OTUs, particularly at the genus level (i.e., *Verticillium*, *Cladosporium*, *Penicillium*, and *Didymosphaeria*), prevail in libraries of infected samples (L2–L6), shrinking the levels of categories that dominate the uninfected (L0) or spiked ones (L1).

All samples (L1–L6) used in this run are considered individual, and any comparison between the different metagenome outcomes is beyond the aims of the present study. Differences observed in the six overall absolute abundance sizes should be considered as stoichiometric and related to the initial PCR product input used for each different sample prior to sequencing. Although, as mentioned in Section 2.6, equimolar amounts of amplicons with the same barcode were pooled, differences may commonly occur due to pipetting accuracy. In multiplexed runs such a situation could occur either among different samples for the same locus (i.e., L2 and L5, Figure 4B) or among different loci for the same sample (i.e., L5, Figure 4A,B). Unless a comparison between samples is the aim, this is a technical issue that does not affect the classification of OTUs for each individual sample.

It is worth noting that major phytopathogens (i.e., *Cladosporium*, *Penicillium*, and *Verticillium*) are detected in the related samples irrespective of the number of read sizes, confirming the microbiological results. Particularly, in the case of samples infected by *Verticillium* (L2–L4) and *Cladosporium*/*Penicillium* (L5), it should be mentioned that nearly half of the aligned reads are reported for these genera, giving an idea of the extent of infection in those samples. Such quantitative data could possibly be exploited further for diagnostic assays. *V. dahliae* is the only OTU reported in the different samples at the species level. Furthermore, it formed a smaller OTU subgroup among all annotations falling in the OTU *Verticillium*. Although this conformed with the BLAST+ filter settings (for species, it was set above 95% of sequence identity), it resulted probably due to a higher representation of more variable sequences that failed to reach this percentage threshold [65]. It is also worth noting that despite the artificial infection of sample L1 with *V. dahliae* DNA, the principal OTU shown for *Verticillium* in this sample only reached the genus level, due to the prevalence of evolutionarily more distant variants [85] and potential sequencing errors [82] that relegate the relevant species’ OTU to lower frequencies and shrink its graphical representation in the abundance plots.

The heat map graphs (Figure 5A–C) present the top 60 OTUs reported regarding their presence and relative abundances in all samples. They provide a more detailed image of the reported OTUs regarding the different loci, and the quantitative representation of every OTU in each sample. Table 3, deduced from the OTUs described in the three different heat maps, gives a summary of the genera and species that are common or not in all three cases.

Of the seven common genera that are reported irrespective of the loci used for OTU classification, four (*Cladosporium*, *Didymosphaeria*, *Penicillium* and *Verticillium*) are phytopathogens commonly infecting olive [86,87,88,89]. Of the remaining three, two (*Aureobasidium* and *Hortaea*) have previously been reported as olive endophytes. *Aureobasidium* was found to inhabit olive leaves, twigs, and branches [87], and was also reported to be associated with grapevine esca disease [46,47,90]. *Hortaea*, a yeast-like fungus, was reported as an endophyte in olive leaves and branches [87]. *Furfurella*, a relatively new genus comprising three species and described to grow on dead branches of Mediterranean fabaceous shrubs was reported for the first time in olive. *Furfurella* species, although they were isolated from bark tissues of different shrub species, were positioned phylogenetically closely to various endophytic fungi [91].

Of the remaining 32 genera reported, either for the ITS- or beta-tubulin-based classification, 20 were previously described as olive phytopathogens and endophytes [27,53,87,88,92]. Among the remaining 12, which were not previously reported as olive-related fungi, there are endophytic genera (*Coleophoma*, *Kwoniella*, and *Paracamarosporium*) [93,94,95], relatively new described species (*Constantinomyces*, and *Neocelosporium*) [96,97], genera with more cosmopolitan fungi found in different matrices (*Scolecobasidium*) [98], fungi which cause post-harvest diseases (*Lambertella*) [99], and genera newly separated or closely linked to others that share high morphological and phylogenetic similarities (*Pseudoseptoria*, *Pseudosydowia*, and *Querciphoma*) [97,100,101].

It should be noted that there are OTUs which are not marked among the top 60 OTUs in a single plex, while they appear in the duplex approach in Table 3**.** The inverse is also observed; OTUs that are present in the single plex column and are absent in the duplex. This is mainly due to the fractionation that characterizes the annotated classes of similar reads, which is evident especially in Figure 5. This fractionation happens because of (a) the inequality of beta-tubulin versus ITS databases in NCBI and the annotation gaps of NCBI taxonomy entries [102], or/and (b) sequence diversification of sample versus NCBI content [103], combined with sequencing errors that drive closely related reads to different annotation classes.

Moreover, as mentioned previously, all samples tested should be considered individual case studies. The heat map plots (Figure 5A–C) illustrate the relative abundances in each sample considering their normalized sum from all samples. A high value of a specific OTU in one of the samples may be mitigated due to lower values in all other samples (e.g., *Paraconyothirium*, and *Paracucurbitaria* in ITS and ITS + beta-tubulin cases). Thus, identification of principal genera should also be evaluated in each individual sample alone to conclude the predominant fungi present, something that is very important when diagnostics rather than community metagenomics is the aim.

The results regarding metabarcoding analysis of infected samples were also compared to the outcome using the BugSeq platform. As seen in Figure 6, the results regarding the predominant genera identified are in line with BLAST+ results and absolute abundances presented in Figure 4C, with four principal genera (*Cladosporium*, *Didymosphaeria*, *Penicilium*, and *Verticillium*) depicted in both analyses.

Our approach showed that the most abundant fungi could be detected to genus and, to a certain extent, to species level, either using single-plex or duplex metabarcoding. In any case, the choice of the most appropriate locus or loci to be used will remain an open question for the less represented species in a sample, until a multiplexed method which would take in account the maximum number of reads and their corresponding sequence identity distributions from all different loci available for each species is developed. It should also be mentioned that the adaptation of our method to the newly available ONT R10.4 flow cells—which allow a very narrow error rate of less than 0.1%—could shift the identification and taxonomy results towards the species level. Furthermore, a future objective would be to test the combination of the ONT-based metabarcoding method with a custom-made database consisting of sequences from fungal species of interest, with the aim to evaluate its use for rapid on-site phytodiagnostics.

## Figures and Tables

**Table 1:** Olive twig samples’ related information.

Plant Sample	Region of Origin	Visual Symptoms	Plant Pathogenic Fungi Isolated	*V. dahliae* PCR Result	Selection for Metabarcoding
1669	Central Greece	yes	*Verticillium dahliae*	positive	yes
1778	Central Greece	yes	*V. dahliae*	positive	yes
1939	Peloponnese	yes	*Alternaria* sp., *Cladosporium* sp., *Aspergillus* sp., *Penicillium* sp.	negative	
2136	Western Greece	yes	*Cladosporium* sp., *Cycloconium oleaginum*, *Penicillium* sp.	negative	
2179	Attica	yes	*Phoma* sp., *Cladosporium* sp., *Penicillium* sp.	negative	yes
2186	Central Greece	yes	*Cladosporium* sp.	negative	
2215	Halkidiki	yes	*Phoma* sp., *Cladosporium* sp., *C. oleaginum*	negative	
3100	Attica	yes	*V. dahliae*	positive	yes
3120	Crete	no	none	negative	yes
3121	Crete	no	none	negative	
3184	Crete	yes	*Phoma* sp.	negative	yes
3186	Crete	no	none	negative	
3869	Peloponnese	yes	*V. dahliae*	positive	
4500	Western Greece	no	none	negative	
4749	Central Greece	no	none	negative	

**Table 2:** Libraries generated according to the SQK-PBK004 four-primer PCR barcoding protocol.

Plant Sample	DNA Sample	Primer Pairs Used in Individual PCRs	Barcode	Library	Sequencing Run
3120	0	ont-ITS1Fngs and ont-ITS4ngs	BR02 ^1^	L0 ^2^	1
		ont-Bt2a and ont-Bt2b			
		ont-ITS1Fngs and ont-LR5			
3120	1 ^3^	ont-ITS1Fngs and ont-ITS4ngs	BR07	L1	2
		ont-Bt2a and ont-Bt2b			
1669	2	ont-ITS1Fngs and ont-ITS4ngs	BR08	L2	
		ont-Bt2a and ont-Bt2b			
1778	3	ont-ITS1Fngs and ont-ITS4ngs	BR09	L3	
		ont-Bt2a and ont-Bt2b			
3100	4	ont-ITS1Fngs and ont-ITS4ngs	BR10	L4	
		ont-Bt2a and ont-Bt2b			
2179	5	ont-ITS1Fngs and ont-ITS4ngs	BR11	L5	
		ont-Bt2a and ont-Bt2b			
3184	6	ont-ITS1Fngs and ont-ITS4ngs	BR12	L6	
		ont-Bt2a and ont-Bt2b			

^1^ Barcode used with the specific DNA sample template and each corresponding primer pair in all individual PCRs. ^2^ Library generated after pooling all individual PCRs accordingly. ^3^ DNA sample “1” is DNA sample “0” artificially inoculated with 1 ng of *V. dahliae* DNA.

**Table 3:** OTUs for genera and species deduced from classifications using separate and combined ITS (I) and beta-tubulin (b-tub (B)) loci.

OTU	ITS (I)	b-tub (B)	I + B	*ol.* ^1^	OTU	ITS (I)	b-tub (B)	I + B	*ol.*
*Alternaria*	+ ^2^		+	(•)	*Penicillium*	+	+	+	(•)
*Aspergillus*		+		(•)	*Phaemoniella*	+			(•)
*Aureobasidium*	+	+	+	(•)	*Phoma*	+			(•)
*Cladosporium*	+	+	+	(•)	*Podosphaera*		+	+	
*Coleophoma*		+	+	(•)	*Pseudorobillarda*		+		
*Coniothyrium*	+		+	(•)	*Pseudocercospora*		+	+	(•)
*Constantinomyces*		+	+		*Pseudoseptoria*	+			
*Didymosphaeria*	+	+	+	(•)	*Pseudosydowia*	+			
*Furfurella*	+	+	+	(•)	*Querciphoma*			+	
*Fusarium*		+		(•)	*Scolecobasidium*		+		
*Hortaea*	+	+	+	(•)	*Septoria*		+		(•)
*Kwoniella*		+			*Stemphylium*	+			(•)
*Lambertella*		+	+		*Stigmina*	+		+	
*Neocatenulostroma*	+			(•)	*Symmetrospora*	+		+	(•)
*Neocelosporium*	+				*Teratosphaeria*		+	+	(•)
*Neodevriesia*	+			(•)	*Trimmatostroma*	+		+	(•)
*Neophaemoniella*		+	+	(•)	*Verticillium*	+	+	+	(•)
*Ochrocladosporium*	+			(•)	*Xenocylindrosporium*	+			(•)
*Paracamarosporium*	+				*C. ramotenellum*	+		+	
*Paraconiothyrium*	+		+	(•)	*P. brevicompactum*		+		
*Paracucurbitaria*	+		+	(•)	*V. dahliae*	+	+	+	(•)

^1^ Bibliography report(s) for OTU as phytopathogen or endophyte in *Olea europea*. ^2^ +: Genus/species reported among top-60 OTUs deduced from each classification. Absence of a cross indicates classification in a higher taxonomical level.

## Data Availability

Fast5 raw sequence data files have been deposited at the NCBI Sequence Read Archive (SRA) under the accession number PRJNA1036004.

## Supplementary Materials

The following supporting information can be downloaded at https://www.mdpi.com/article/10.3390/jof9111119/s1. Supplementary Figure S1. Agarose gel electrophoresis of PCR amplicons from different olive twigs’ samples using Verticillium dahliae specific primers. L: GeneRuler 100-bp DNA ladder (Thermo Fisher Scientific, Waltham, MA, USA) 1669–4749: olive twigs’ samples as described in Table 1; H_2_O: negative control; Vd: Verticillium dahliae BPIC 2696 isolate DNA used as positive control. Supplementary Figure S2. Agarose gel electrophoresis of PCR amplicons from different fungal species isolates using different primer pairs: (a) Amplicons using the ont-ITS1Fngs & ont-ITS4ngs; (b) Amplicons using the ont-ITS1Fngs & ont-LR5; (c) Amplicons using the ont-Bt2a & ont-Bt2b; (d) Amplicons using the ont-CDM5 & ont-CDM6. L: GeneRuler 100-bp DNA ladder (Thermo Fisher Scientific, Waltham, MA, USA) Pn: Penicillium commune; Cl: Cladosporium sp.; Asp: Aspergillus niger; Al: Alternaria alternata; Ph: Phoma incompta; Vd: Verticillium dahliae; H2O: negative control. Supplementary Figure S3. Instance file report for ONT run ID_292883 regarding library L0. Read counts regarding library L0 are shown in the Reads per barcode section as barcode 02. Total reads analyzed, total yield, average quality score, and sequence lengths distribution are shown. Supplementary Figure S4. Instance file report for ONT run ID_367816 regarding libraries L1-L6. Read counts regarding each of the six libraries are shown in the Reads per barcode section as barcodes 07 to 12 for libraries L1 to L6 respectively. Total reads analyzed, total yield, average quality score, and sequence lengths distribution are shown. Supplementary Figure S5. Distributions of lengths for raw reads and their BLAST+ matches for samples L1–L6. Histograms depict the distributions of the lengths of raw reads (left), and their best matches (right) according to BLAST+ analysis. Lengths smaller than 250 bp were excluded from best matches panel (right).
