# Supplementary figures and images for: Nanopore-Sequencing Metabarcoding for Identification of Phytopathogenic and Endophytic Fungi in Olive (Olea europaea) Twigs
Source: J Fungi (Basel). 2023 Nov 18;9(11):1119. doi: 10.3390/jof9111119 (PMC10672464; doi:10.3390/jof9111119)

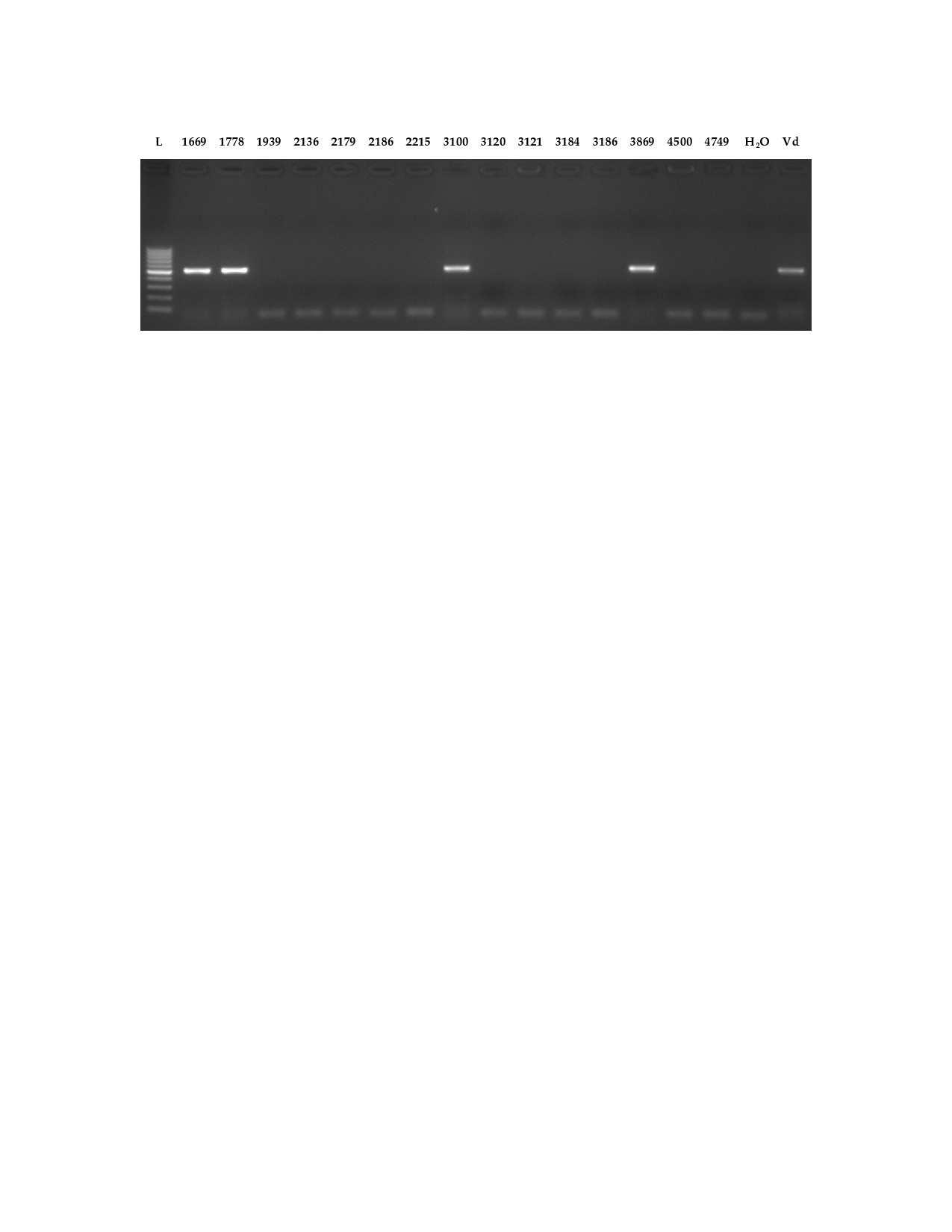

Supplement: Supplementary file 1 [file jof-09-01119-s001.zip › Supplementary Figure S1.jpg]

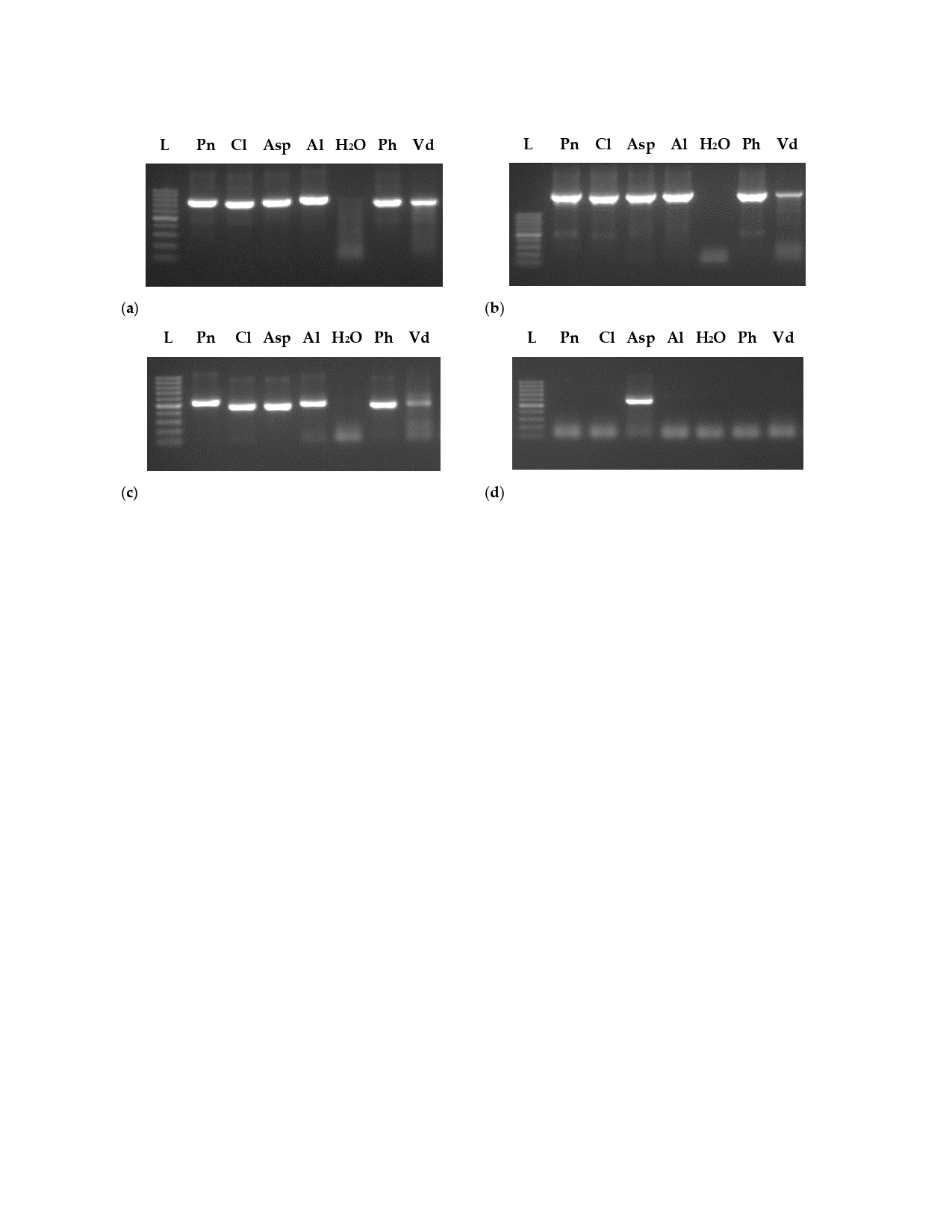

Supplement: Supplementary file 1 [file jof-09-01119-s001.zip › Supplementary Figure S2.jpg]

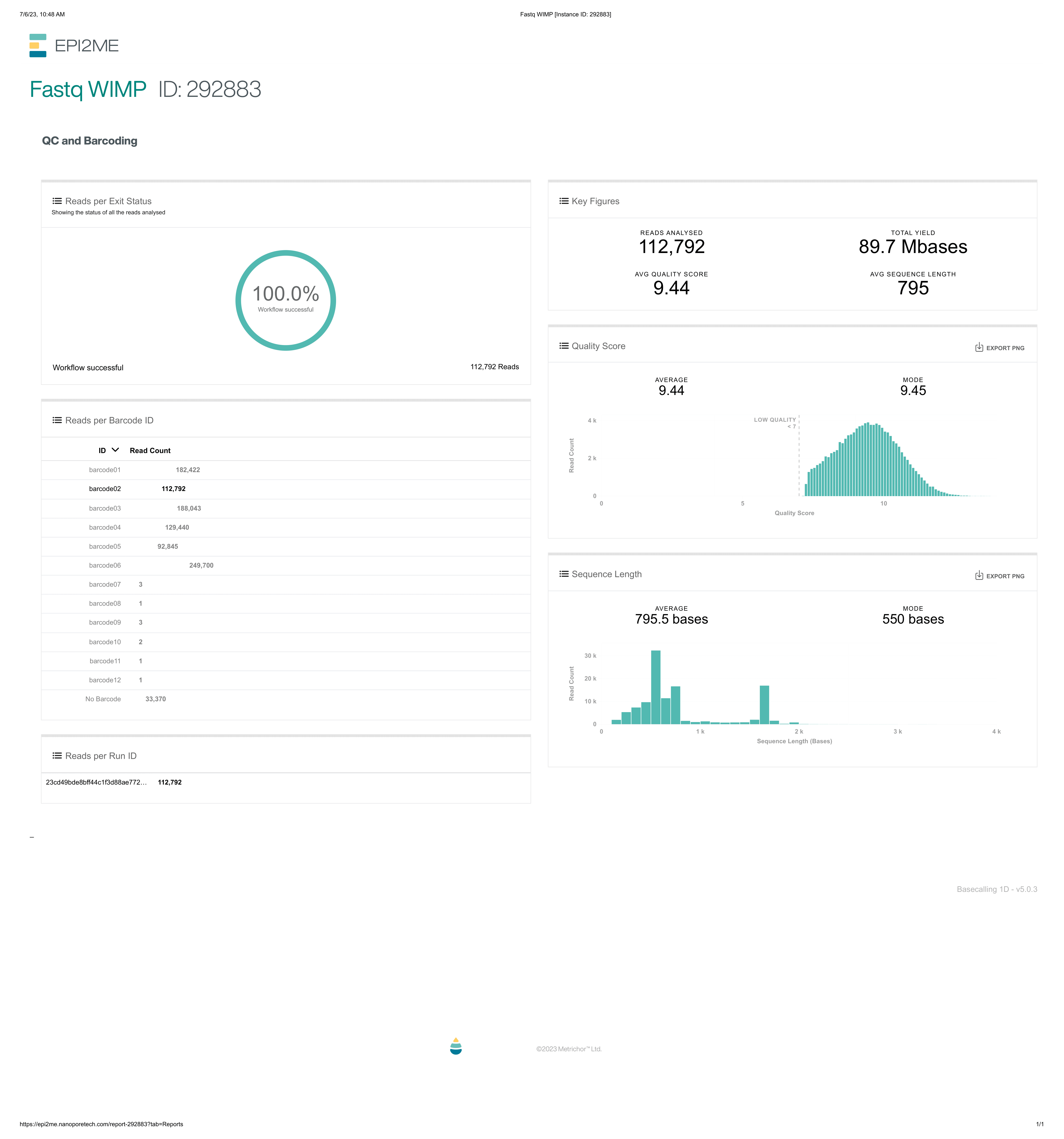

Supplement: Supplementary file 1 [file jof-09-01119-s001.zip › Supplementary Figure S3.png]

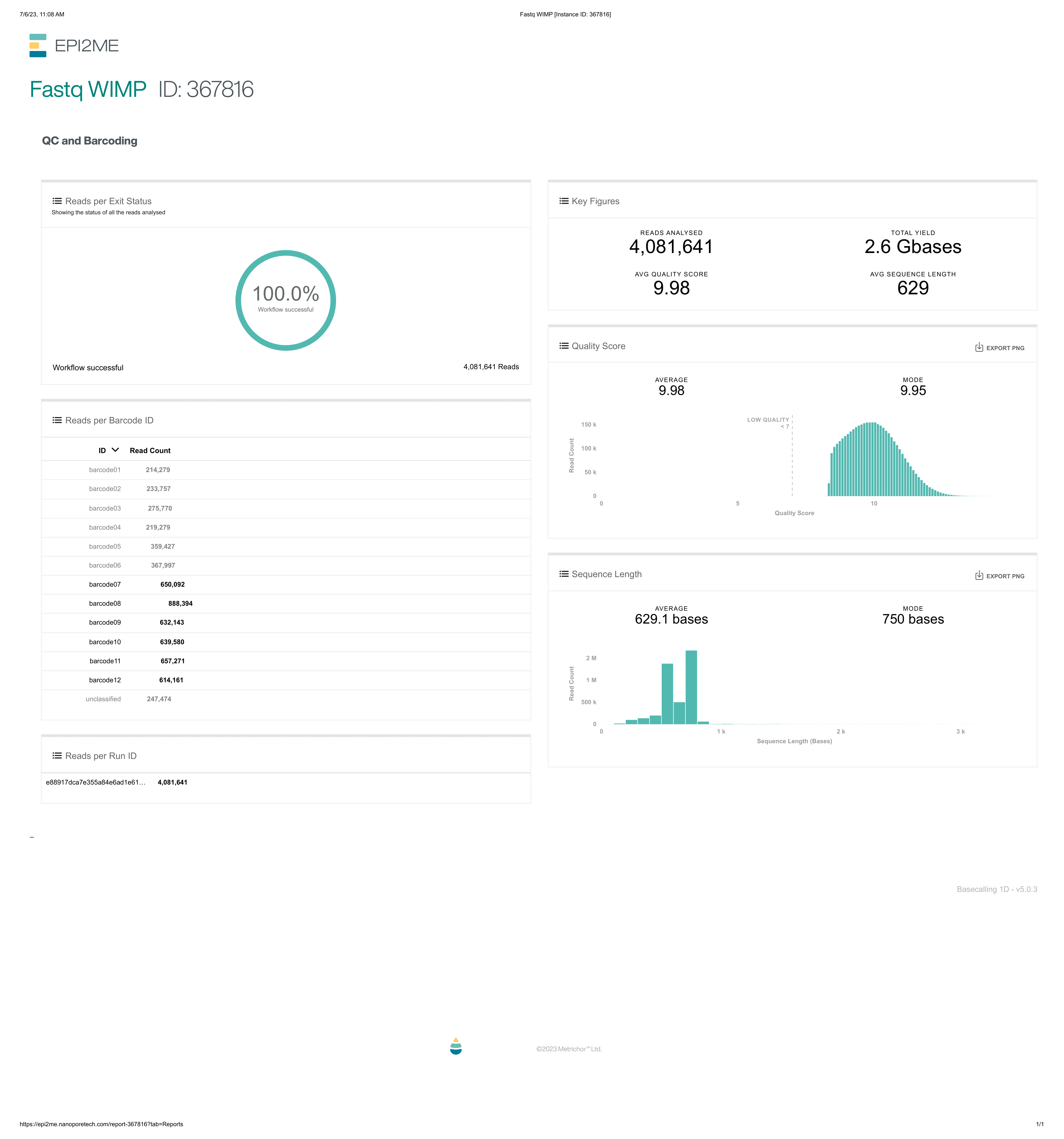

Supplement: Supplementary file 1 [file jof-09-01119-s001.zip › Supplementary Figure S4.png]

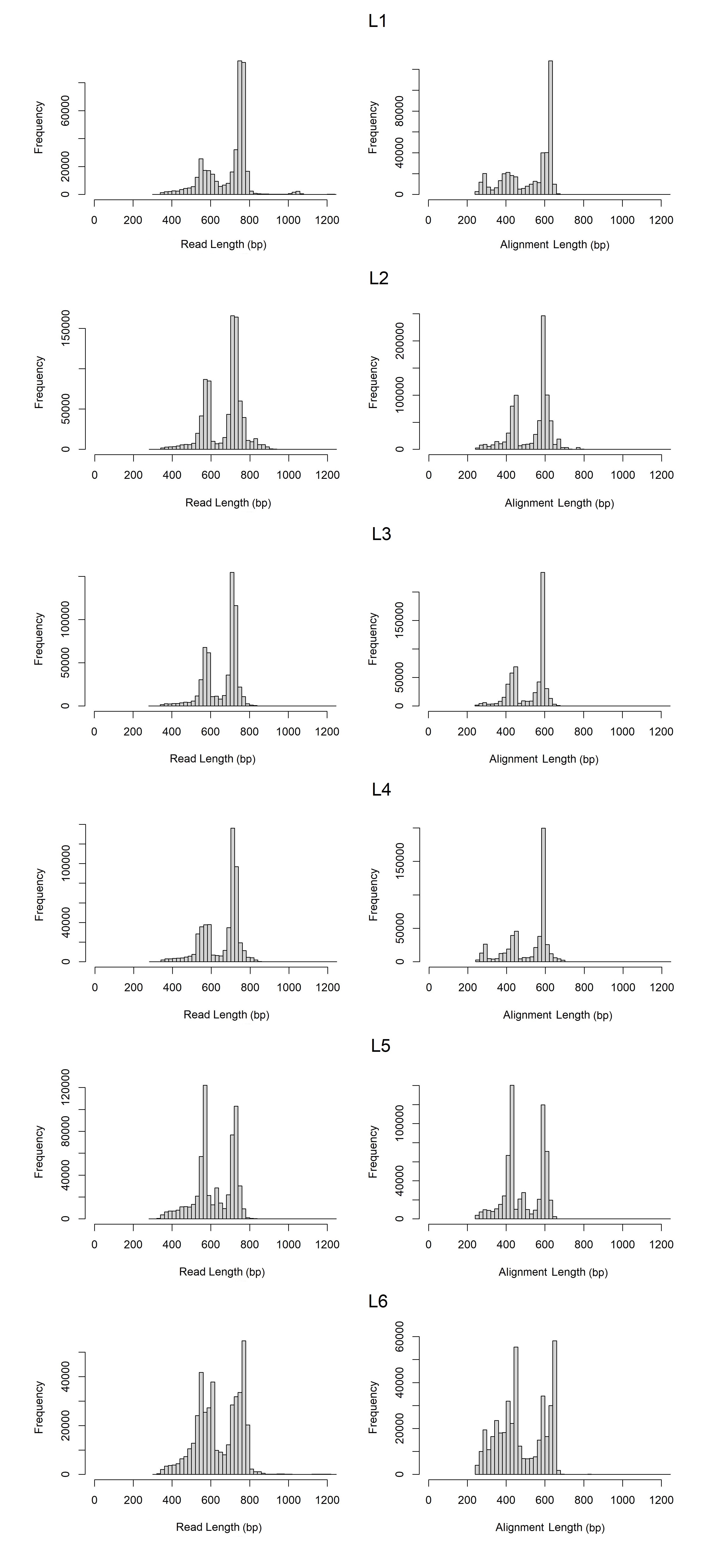

Supplement: Supplementary file 1 [file jof-09-01119-s001.zip › Supplementary Figure S5.jpg]
